# Comparable efficacy of autogenous tooth-derived grafts and xenografts in post-extraction osseous defects: a split-mouth randomized trial

**DOI:** 10.1007/s00784-025-06458-3

**Published:** 2025-07-23

**Authors:** Phaichaya Laiamnuay, Teeranut Chaiyasamut, Rui Zhang, Dutmanee Seriwatanachai, Kornkamol Kretapirom

**Affiliations:** 1 Department of Oral and Maxillofacial Surgery, Faculty of Dentistry, Mahidol University, Bangkok, 10400 Thailand; 2 Department of Periodontics, School and hospital of stomatology, Kunming Medical University, Kunming, 650106 China; 3 Department of Oral Biology, Faculty of Dentistry, Mahidol University, Bangkok, 10400 Thailand; 4 Department of Oral and Maxillofacial Radiology, Faculty of Dentistry, Mahidol University, Bangkok, 10400 Thailand

**Keywords:** Alveolar ridge preservation, Bone regeneration, Alveolar ridge preservation, Fractal analysis, Bone substitute materials

## Abstract

**Objective:**

To evaluate the efficacy of autogenous tooth substitute graft (ATSG) prepared from autogenous third molars compared to xenograft in reducing osseous defects at the distal aspect of the second molars after impacted mandibular third molars removal.

**Materials and methods:**

A randomized, double-blind, split-mouth clinical trial was conducted with 14 patients (aged 20–30 years) with bilateral mesioangular or horizontal impacted mandibular third molars. Following extraction, one socket received autogenous tooth-derived graft material (experimental site) prepared using a dentine grinder while the contralateral socket received xenogenic bone substitute (control site). Clinical parameters (probing depth, mobility, pain score, healing) and radiographic outcomes (mean gray value, fractal dimension, bone integration, dimensional changes) were evaluated pre-operatively and at 2 weeks, 3 months, and 6 months post-operation.

**Results:**

Both groups showed similar clinical outcomes with no statistically significant differences in probing depth, mobility, pain scores, or healing. Radiographically, the mean gray value decreased by 4.62% in the autogenous group and 2.19% in the xenogeneic group at 6 months, though the differences were not statistically significant. Notably, by 6 months, total bone integration was observed in 64.29% of autogenous grafting sites compared to 57.14% of xenogeneic grafting sites. Fractal dimension analysis further suggested distinctive trabecular patterns between the two materials. Both materials demonstrated excellent biocompatibility with comparable dimensional stability; no significant differences were found in vertical or horizontal dimensional changes between the groups.

**Conclusions:**

Autogenous tooth-derived bone substitute demonstrates comparable clinical and radiographic outcomes to xenograft when used for alveolar ridge preservation following mandibular third molar extraction, suggesting it may be a viable alternative to conventional xenografts.

**Clinical relevance:**

ATSG offers a chairside-prepared autogenous grafting option that can be completely produced in under twenty minutes, eliminating the costly and time-consuming laboratory processing needed for other materials while maintaining comparable clinical efficacy to the commercial xenografts.

**Supplementary Information:**

The online version contains supplementary material available at 10.1007/s00784-025-06458-3.

## Introduction

The third molars are the last permanent to erupt chronologically in the oral cavity. By the time of their emergence, the maxilla and mandible have typically completed their development, which can limit the available space for their eruption. As a result, most third molars need to be extracted. The prevalence of periodontal bone loss distal to the adjacent second molar was higher in individuals with mesioangular and horizontal impactions [1]. A two-year follow-up study examining periodontal healing after third molar surgical removal reported that 43.3% of adjacent second molars exhibited pocket depths exceeding 7 mm, while 32.1% demonstrated bone loss greater than 4 mm [2].

To address alveolar bone defects following tooth extraction, researchers have developed various bone substitute biomaterials. Nevertheless, autologous bone remains the gold standard for bone augmentation procedures due to its unique combination of osteogenic, osteoconductive, and osteoinductive properties [3]. However, autologous bone has disadvantages including donor site morbidity, limited availability, and additional surgical complexity. There is also a clinical need for slow-resorbing bone substitutes that maintain space during the extended healing process. In 2010, Kim et al. introduced an innovative approach by successfully utilizing demineralized dentine from the patient’s extracted teeth as a bone substitute material to promote bone regeneration [4]. Study have shown that autogenous demineralized dentin matrix derived from extracted teeth, when grafted into extraction sockets for vertical augmentation, demonstrates comparable effectiveness to inorganic bovine bone. Both materials resulted in favorable wound healing, similar level of implant stability, and histologically confirmed new bone formation [5].

In 2021, Khanijou et al. published a study of physicochemical and biological properties of autogenous tooth substitute graft (ATSG)– a complete non-demineralized whole tooth. X-ray diffraction analysis (XRD) demonstrated the structural similarity between dental tissues and alveolar bone. The inorganic component of the tooth consists of hydroxyapatite (HA) with smaller amounts of tricalcium phosphate (TCP), amorphous calcium phosphate (ACP), and octacalcium phosphate (OCP). The organic components include type I and type III collagens, along with growth factors such as BMP-2, IGF-II, and TGF-β, which play crucial roles in bone remodeling [6–9]. ATSG showed no microbial growth following the chemical decontamination, and its biocompatibility makes it suitable for bone augmentation procedures [6, 10].

Although numerous studies have investigated the use of autogenous tooth (AutoBT) or demineralized dentine for osseous defect augmentation due to their autogenous properties and proven efficacy, the preparation of the demineralized dentine graft is time-consuming. This process requires specialized laboratory treatment that may extend from several days to a week [11]. In contrast, ATSG can be prepared chairside using a tooth grinding machine in less than twenty minutes allowing for immediate use of fresh grafting material during the same dental visit.

This clinical trial therefore aimed to evaluate the upcycling of the whole impacted mandibular third molars to reduce bone defects at the distal aspect of the mandibular second molars following surgical removal procedures. The study investigated a split-mouth design to compare ATSG with xenograft placement at the surgical site. We evaluated new bone formation after augmentation through multiple methods: assessing the radiographic mean gray value of the grafted area, measuring dimensional changes in alveolar bone, and performing fractal analysis.

## Materials and methods

### Ethical statement

This study was a clinical trial. ATSG was obtained from the segmented impacted tooth, processed according to the tooth grinder manufacturer’s instructions and grafted into each subject individually, manufacturer’s instructions https://www.kometabio.com. All methods and experimental protocols were approved and carried out in accordance with the Faculty of Dentistry/Faculty of Pharmacy, Mahidol University, Institutional Review Board (COE.No.MU-DT/PY-IRB 2022/057.0711). This study was registered in the Thai Clinical Trials Registry (TCTR20221216003).

### Study design

This study was designed to be a randomized, split-mouth clinical trial. The study was conducted at the Oral and Maxillofacial Surgery Clinic and Oral and Maxillofacial Radiology Clinic, Faculty of dentistry, Mahidol University, Thailand.

**Inclusion criteria**

- Male or female aged between 20– 30 years
- Patients with bilateral mesioangular or horizontal impacted mandibular third molars with present second molars. The impacted teeth could be of either type but must be symmetrical in each subject
- No pathological lesion involved either the mandibular second and third molars or surrounding bone

- Patients demonstrating good oral hygiene according to the Simplified Oral Hygiene Index [12], [13]
- Cooperative patients capable of following instructions
- Patients who consented to the use of porcine xenogeneic material

**Exclusion criteria**

- Patients with systemic diseases or medical conditions that might impair wound healing (e.g., diabetes mellitus, immunocompromised conditions)
- Patients with existing active infection at either mandibular third molar site
- Patients who smoke
- Patients with orthodontic appliances

### Sample size

The sample size was calculated according to a formula below which was used in many clinical research [14]. Twenty percent was added for attrition during the study, therefore n = 15 was used in the study.

$$\begin{array}{cc}n=\frac{\left(Z_{1-{\displaystyle\frac\alpha2}}++Z_{1-\beta}\right)^2\sigma^2}{\triangle^2}&\begin{array}{c}SD.\left(\sigma\right)=1.78,\;Delta\;\left(\triangle\right)=1.48\\Alpha\;\left(\alpha\right)=0.05,\;Z\left(0.975\right)=1.959964\\Beta\;\left(\beta\right)=0.20,\;Z\;\left(0.800\right)=0.841621\end{array}\end{array}$$

### Participants

**Fig. 1:**
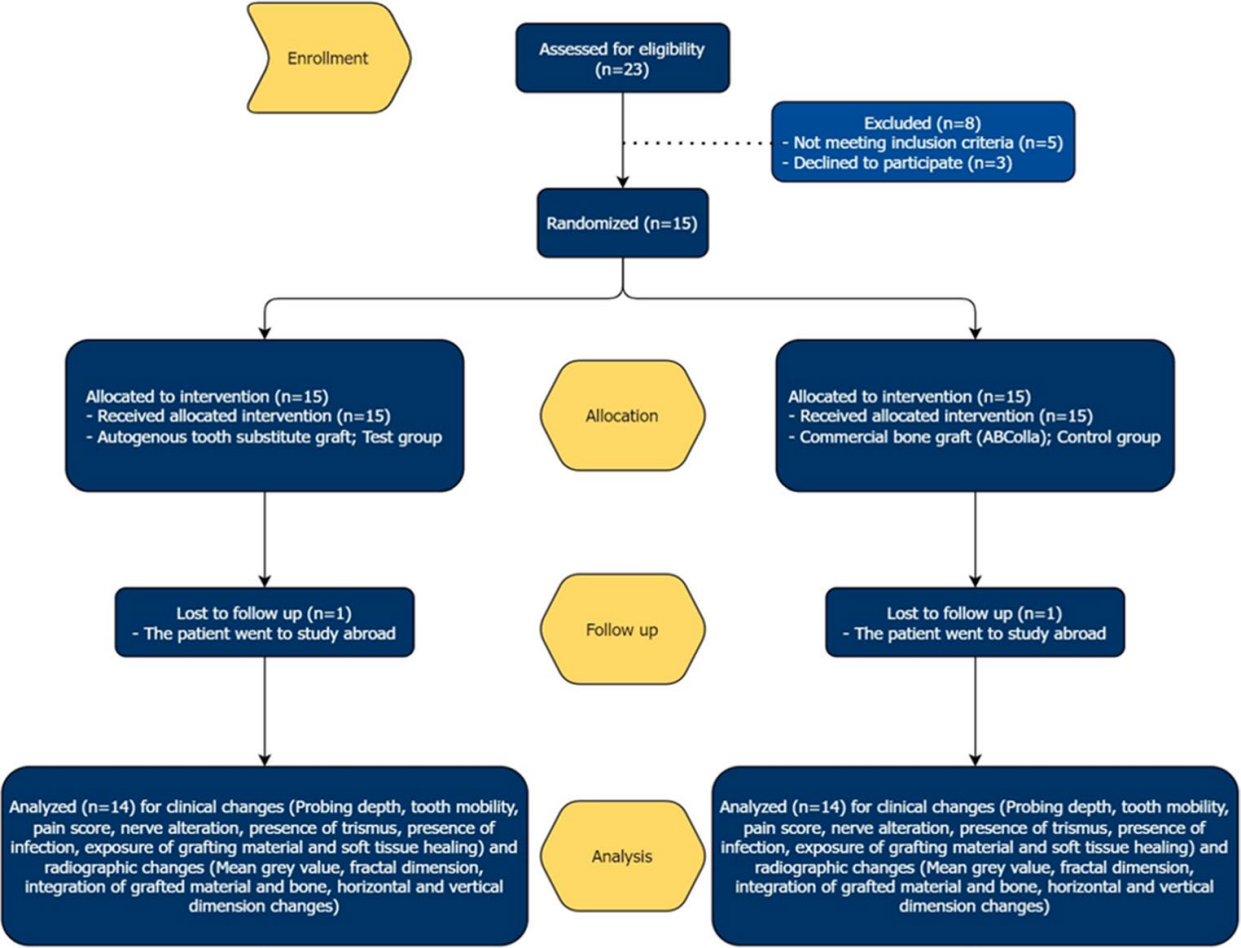
CONSORT flowchart of the clinical study design

Twenty-three patients from the Oral and Maxillofacial Surgery Clinic and Oral and Maxillofacial Radiology Clinic, Faculty of dentistry, Mahidol University, were initially enrolled in the study. Eight patients were excluded due to exclusion criteria. Fifteen patients who met all eligibility criteria were selected to proceed with the following experiments, as illustrated in figure 1. The researcher conducted comprehensive medical and initial dental assessments to collect all necessary data. Prior to obtaining informed consent, all participants were provided detailed information about the study, including its purposes, surgical procedures, and potential complications.

### Randomization and blinding

This study was a double-blind trial, as neither the participants nor the primary investigator knew which site was experimental and which was control. The sites were randomly assigned by program available online (research randomizer) before the grafting step, whether for ATSG or xenograft.

### Patient instruction

Each patient was fully informed about the study, including its purposes, surgical procedure details, and possible complications, before signing the informed consent. After the surgical procedure, Amoxicillin (500 mg), 20 capsules, Sig. 1 cap p.o. t.i.d.; Ibuprofen (400 mg), 10 tablets, Sig. 1 tab p.o. t.i.d. p.c.; and Paracetamol (500 mg), Sig. 1 tab p.o. p.r.n for pain or fever q.6.h, were prescribed. Post-operative instructions were explained by the surgeon. Stitches were removed two weeks post-operation, followed by follow-up visits at three and six months post-operation.

### Surgical procedure

The surgical removal of mandibular third molars at both experimental and control sites was performed using identical flap design and surgical techniques. During the procedure minimal buccal bone osteotomy was done just below the height of contour of the impacted tooth. At the experimental site, the extracted tooth was thoroughly cleaned of soft tissue and debris from its crown and root surfaces, followed by copious irrigation with normal saline solution. After drying with sterile gauze, the tooth was processed in a Smart Dentin Grinder™ (KometaBio Inc., USA). The ground tooth material was then sieved to obtain particles between 300-1,200 μm in size. These particles underwent chemical treatment in two sequential solutions: first in 0.5 M NaOH with 30% ethanol (v/v) for 5 minutes, then in Dulbecco's Phosphate Buffered Saline for 2 minutes. The resulting autogenous tooth-derived graft material was rinsed with normal saline solution, dried with sterile gauze, and placed in the extraction socket at the experimental site. At the control site, porcine bone substitute material (ABCcolla^® ^Bone Graft, ARCO Biomedical Co., Ltd., Taiwan) was placed in the extraction socket. Both sites were sutured in primary closure manner using silk sutures, without any membrane or adjunctive material placement. All procedures, from local anesthesia administration to wound closure, were performed by a single oral and maxillofacial surgeon (T.C.).

### Evaluation

In the clinical evaluation, the data including probing depth (PD) of three reference points - disto-buccal, mid-distal, and disto-lingual at distal root and the mobility of the second mandibular molar (MOB) were collected at pre-operation, 2 weeks post-operation, 3 months and 6 months post-operation. The clinical findings including pain score (PS), presence of trismus (T), presence of surgical site infection (INF), nerve alteration (N), exposure of grafting material (EXP), and soft tissue healing (EHS) [15] were collected 2 weeks, 3 months and 6 months post-operation. 

For the radiographic evaluation, standard periapical films were recorded pre-operatively and, 2 weeks, 3 months, and 6 months post-operation. Each intraoral radiography was performed under the same protocol using a holder device (Rinn centering device, Dentsply Ltd, Weybridge, UK). The anterior border of the intraoral film was positioned at the mesial aspect of the crown of the first mandibular molar. The distal alveolar bone and root apex of the distal root of the second mandibular molar must be seen in the radiograph. Each intra-oral radiographic examination was performed with Planmeca Intra (PLANMECA Oy, Helsinki, Finland) x-ray unit under identical exposure parameters including 66 KV, 8 MA, and exposure time of 0.25 sec., digitized and saved in Joint Photographic Experts Group format (JPEG).

The mean gray value (MGV) which implies to bone density at the grafted area was measured on the selected region of interest (ROI) as described in supplemental figure 1 (fig. S1). Briefly, the ROI was created by drawing a box approximating the periodontal space of the distal root of the mandibular second molar. In the supero-inferior dimension, the vertical border of the box extended from the cemento-enamel junction (CEJ) to the apex of the distal root of the mandibular second molar, with a width of 2 mm. using ImageJ 2.1.0 software (GNU General Public License). The program calculated the MGV which reflects bone density in the ROI, and the values were compared in each interval of time. In fractal analysis, the same ROI as in MGV analysis was used. Then gaussian blur was used to remove brightness variations due to overlying soft tissues and varying thickness of bone. The resulting image was then subtracted from the original image. Then the bone marrow spaces and trabeculae were discriminated from each other with the addition of 128 gray value to each pixel location. After binarization of the ROI, bone marrow spaces and trabeculae were outlined. The noise of the resulting image was eliminated with erosion, and the outlines of the structures were emphasized using dilation. The image was inverted to make the trabeculae black and bone marrow spaces white in skeletonization. The FD of the trabecular structure was calculated via ImageJ 2.1.0 software and the values were compared in each interval of time.

Cone beam computed tomography (CBCT) images were obtained using the 3D Accuitomo (J Morita Mfg. Corp., Kyoto, Japan). The scan parameters were set to 90 kV, 5 mA, with a field of view (FOV) of 6 cm x 6 cm, and a voxel size of 0.125 mm. Data from the scans were analyzed using i-Dixel 2.0 software (J Morita Mfg. Corp., Kyoto, Japan). CBCT scans were conducted pre-operation, at 3 months, and at 6 months post-operation. CBCT was employed to evaluate grafting material and bone integration (INT), vertical bone dimensional change (ΔV), and horizontal dimensional bone change (ΔH). The criteria for evaluating grafted material and bone integration are described in the supplemental data. The INT, ΔV, and ΔH were measured at reproducible reference points and lines, as shown in supplemental data, supplemental figure 2 and 3 respectively and comparisons of the measurements from each film taken at different time intervals were made. To minimize examiner bias during the radiographic data analysis, intra- and inter-examiner reliability calibrations were conducted. For intra-examiner reliability, the primary investigator (P.L.), collected data from each participant three times over approximately one week to reduce reliance on memory regarding individual case characteristics. For inter-examiner reliability, 10% of cases were randomly selected and independently analyzed by a second investigator (K.K.). Calibration between the two investigators yielded an intraclass correlation coefficient (ICC) of 0.90, indicating excellent agreement.

### Statistics

The data were analyzed using IBM SPSS Statistics for Windows, Version 27.0. Armonk, NY: IBM Corp and RStudio 2023 version 12.0. In the same group of data collecting at different periods of time including PD, MGV, FD, ΔV and ΔH, two-way repeated ANOVA was used to analyze interaction between each variable and time. Mean +/- SD of MGV, FD, PD, vertical and horizontal dimensional change were analyzed for any significant change at p-value < 0.05. Grafted bone margin integration (Int) and mobility were analyzed with the McNemar test. Pain score (PS) and soft tissue healing (EHS) were analyzed with the Wilcoxen test.

## Results

A total of 15 patients were enrolled in the study, with one patient excluded due to overseas relocation, resulting in 14 patients for final analysis. Demographic characteristics of all participants are presented in Table 1. Based on Pell and Gregory's classification, the impacted mandibular third molars showed equal distribution between mesioangular (MA) and horizontal angulation (HA). Depth B was the most prevalent, followed by depths A and C. No significant differences were observed in pre-operative probing depths across measurement sites of mandibular second molars.

**Table 1:** Demographic data of the participants in ATSG and xenogeneic bone graft materials

		Autogenous	Xenogeneic	*p*-value
Age (years), mean +/- SD		23.14 +/- 2.74		-
Gender	Male, n (%)	5 (35.70%)		-
	Female, n (%)	9 (64.30%)		
Angulation	MA, n (%)	7 (50.00%)	7 (50.00%)	1.000^a^
	HA, n (%)	7 (50.00%)	7 (50.00%)	
Depth	A, n (%)	2 (14.30%)	2 (14.30%)	0.317 ^a^
	B, n (%)	10 (71.40%)	11 (78.60%)	
	C, n (%)	2 (14.30%)	1 (7.10%)	
Location	Right, n (%)	6 (42.90%)	8 (57.10%)	0.791 ^a^
	Left, n (%)	8 (57.10%)	6 (42.90%)	
Probing depth (mm), mean +/- SD	Disto-buccal	4.36 +/- 2.21	3.79 +/- 1.12	.319^b^
	Mid-distal	3.43 +/- 0.646	3.79 +/- 1.58	0.418 ^b^
	Disto-lingual	3.93 +/- 1.33	3.64 +/- 1.22	0.414 ^b^
Mobility (degree);	0 degree, n (%)	14 (100.00%)	13 (92.90%)	-
	1 degree, n (%)	0 (0.00%)	1 (7.10%)	

SD, standard deviation; n, number; mm, millimeter; a, p-value from McNemar test; b, p-value from Paired Samples t test

Different letter indicates statistically significant differences within the same graft

### Clinical outcomes

PD showed no statistically significant differences within or between groups. Both groups exhibited peak PD values at 2 weeks post-operation, followed by gradual decrease over time (table S1). One patient in the xenogeneic group presented with first-degree mobility pre-operatively. Both groups equally showed 21.40% of first-degree mobility at 2 weeks. At 3 months, there was 35.70% with first-degree mobility in autogenous group and 28.60% in xenogeneic group. At 6 months, mobility was absent in both groups. No second- or third-degree mobility was observed throughout the study (table S2). Median pain score (PS) and early healing score (EHS) showed no statistically significant differences between groups (table 2). Complications included one case of inferior alveolar nerve injury. There were two cases (7.14%) of local infection—one from each group—and two cases (7.14%) of wound dehiscence with grafting material exposure, also one from each group, observed at 2 weeks post-operation. All complications resolved with favorable secondary healing. No cases of trismus were reported.

**Table 2:** Pain score and early healing score (EHS)after operation for 2 weeks

			Graft Autogenous			Graft Xenogeneic				*p*-value		
Pain Score, median(min-max)			1 (0–6)			0.5(0–4)				.194^a^		
EHS, median(min-max)			10 (2–10)			10(4–10)				.916^a^		

a, *p*-value from Wilcoxon test

### Radiographic outcomes

**Fig. 2:**
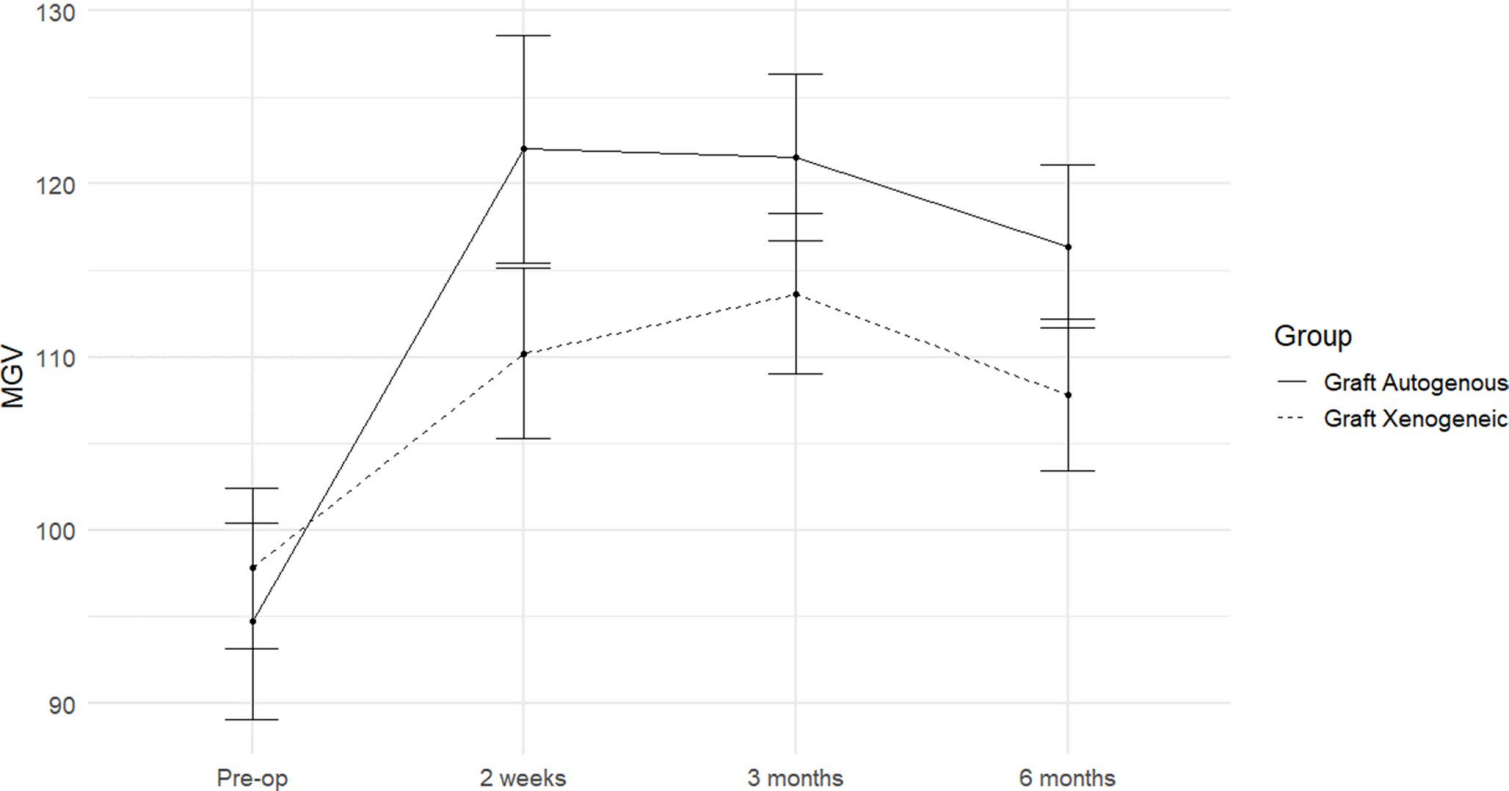
Average of radiographic mean gray value (MGV) at pre-op, 2 weeks, 3 months, 6 months after grafting with autogenous or xenogenic materials

**Fig. 3:**
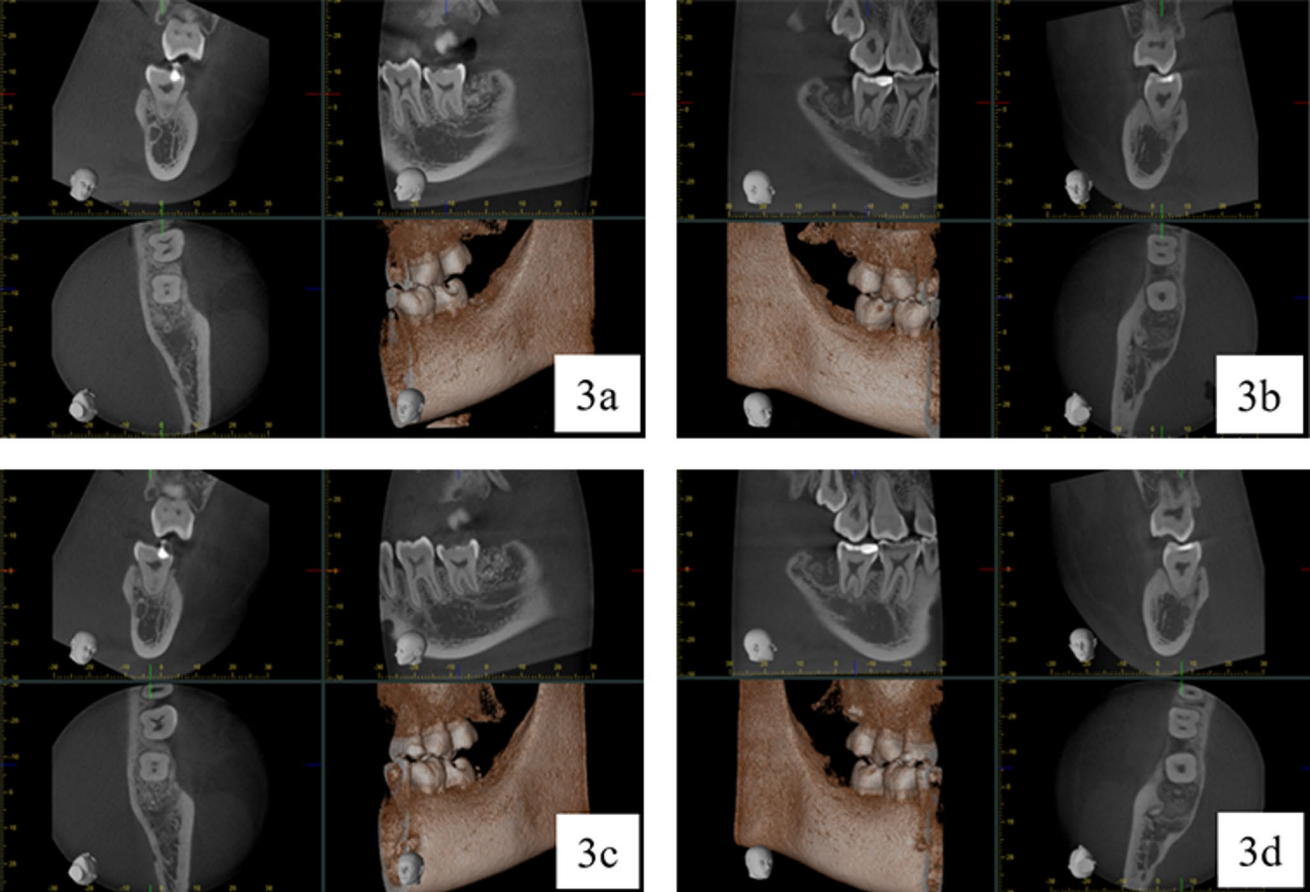
CBCT analysis revealed partial osseointegration of ATSG at 3 months post-operatively (42.86%); (3a), while the xenogenic graft demonstrates no osseointegration during the same period (28.57%); (3b). At the 6-month follow-up, complete osseointegration of the ATSG is observed (64.29%); (3c), whereas the xenogenic graft continues to show no evidence of osseointegration (14.29%);(3d)

Two-way repeated ANOVA revealed that both time and grafting material type influenced MGV. However, neither factor affected FD, and no interaction was observed between time and graft type. In the autogenous group, MGV increased significantly following graft placement due to material properties, then decreased by 4.63% at 6 months post-operation. While the xenogeneic group showed a slow increase in MGV, reaching its peak at 3 months post-operation, before decreasing by 2.19% at 6 months post-operation. No significant differences in MGV were found within or between groups (table 3). Figure 2 demonstrates the MGV graph between the autogenous and xenogenic groups in time difference. The MGV in the autogenous group (solid line) increased significantly in the beginning then gradually decreased. In contrast, MGV in the xenogeneic group (dashed line) gradually increased to its peak at 3 months post-operation before declining. FD values were lower in the autogenous group compared to the xenogeneic group across all time points, though differences were not statistically significant (table 4). At 3 months post-operation, the autogenous group showed total integration in 50% of sites, partial integration in 42.90%, and no integration in 7.10%. The xenogeneic group predominantly showed partial integration (57.10%), followed by no integration (28.60%) and total integration (14.30%). By 6 months, total integration increased to 64.30% of autogenous sites and 57.11% of xenogeneic sites, with partial integration equally present in both groups (28.60%) (table 5). Figure 3 illustrates the CBCT analysis showing osseointegration progression in some representative samples from the ATSG and xenogeneic graft groups at 3 and 6 months post-operatively. Two-way repeated ANOVA indicated no influence of time or grafting material type on ΔV or ΔH, with no interaction between factors. Both ΔV and ΔH measurements were lower in the autogenous group compared to the xenogeneic group, though differences were not statistically significant (table 6 and 7).

**Table 3:** Mean +/- SD of boneradiographic mean gray value (MGV)

	Graft Autogenous	Graft Xenogeneic	p-value
Pre-op	94.76 +/- 21.29^a^	97.82 +/- 17.37^a^	.546
2 weeks	122.03 +/- 24.61^b^	110.23 +/- 18.41^a^	.016
3 months	121.54 +/- 18.12^b^	113.66 +/- 17.39^a^	.040
6 months	116.39 +/- 17.59^b^	107.81 +/- 16.37^a^	.012

Different letter indicates statistically significant differences within the same graft

**Table 4:** Mean +/- SD of fractal dimension of trabeculae at the grafting areas

	Graft Autogenous	Graft Xenogeneic	*p*-value
Pre-op	1.05 +/- 0.09^a^	1.05 +/- 0.09 ^a^	0.994
2 weeks	1.06 +/- 0.07 ^a^	1.07 +/- 0.07 ^a^	0.908
3 months	1.08 +/- 0.06 ^a^	1.11 +/- 0.04 ^a^	0.070
6 months	1.08 +/- 0.06 ^a^	1.10 +/- 0.02 ^a^	0.403

Different letter indicates statistically significant differences within the same graft

**Table 5:** Bone integration score from different grafting areas at 3 and 6 months after operation

Time	Graft Autogenous	Graft Xenogeneic	*p*-value
3 months			
no bone integration	1(7.14%)	4(28.57%)	.149^a^
Partial bone integration	6(42.86%)	8(57.14%)	
100% bone integration	7(50.00%)	2(14.29%)	
6 months			
no bone integration	1(7.14%)	2(14.29%)	.607^a^
Partial bone integration	4(28.57%)	4(28.57%)	
100% bone integration	9(64.29%)	8(57.14%)	

a, p-value from McNemar test

**Table 6:** Mean +/- SD of Δ vertical bone dimension

	Graft Autogenous	Graft Xenogeneic		*p*-value	
ΔV_1_	-3.86 +/- 2.39^a^	-4.11 +/- 2.42 ^a^	0.709		
ΔV_2_	-3.67 +/- 2.57 ^a^	-3.87 +/- 2.36 ^a^	0.789		

Different letter indicates statistically significant differences within the same graft

ΔV_1_ is a vertical dimension change from pre-operation and 3 months post-operation

ΔV_2_ is a vertical dimension change from 3 to 6 months post-operation

**Table 7:** Mean +/- SD of Δ horizontal bone dimension

	Graft Autogenous	Graft Xenogeneic	*p*-value
ΔH_1_	-1.60 +/- 1.55^a^	-1.79+/- 3.37 ^a^	0.226
ΔH_2_	-1.57 +/- 1.44 ^a^	-2.19 +/- 2.92 ^a^	0.498

Different letter indicates statistically significant differences within the same graft

Δ H_1_ is a horizontal dimension change from pre-operation and 3 months post-operation

Δ H_2_ is a horizontal dimension change from 3 to 6 months post-operation

## Discussion

This study represents the first randomized, split-mouth clinical trial comparing the efficacy of ATSG and xenogeneic material in reducing osseous defects. While autogenous tooth substitute grafts have been utilized for decades, most published studies focused on demineralized dentine matrix preparation. Our study is distinctive in utilizing non-demineralized whole tooth, which streamlines the preparation process and maximizes the utility of every part of the extracted tooth.[4].

MGV, indicating x-rays absorption which represents the radiologic density in certain tissue of selected region. It directly relates to the degree of bone formation. In gray value calculation, every pixel obtains a value between 0 and 255 in which 0 stands for black - low radiologic density and 255 for white - total x-ray absorption [16]. Our findings demonstrated no significant differences in bone formation between the two grafting materials. This suggests comparable efficacy between ATSG and xenogeneic materials in promoting osseous regeneration. Fractal analysis, a method for studying the microarchitecture of bone trabeculae, has found various applications in dentistry. These applications include assessing trabecular structure of mandibular condyles in patients with temporomandibular disorders, characterizing sialography, and evaluating mandibular osseous healing after orthognathic surgery [17]. Therefore, a measurement of FD serves as a reliable predictor of dental implant osseointegration, providing objective quantitative evidence of alveolar bone healing that surpasses visual assessment alone [18, 19]. Our study demonstrated a gradual increase in FD in the autogenous group, aligning with findings from Soylu et al.'s study analyzing fractal dimension during osseointegration following dental implant placement. Previous research on fractal analysis in orthognathic surgery cases similarly concluded that fractal dimension values between osteotomy lines increased over time. Mu et al. reported significant increases in mean FD from 1.42 ± 0.05 to 1.43 ± 0.05 after one year, correlating with bone remodeling processes. The absence of significant changes in mean FD in our study likely reflects our shorter follow-up period compared to previous research [18, 20]. CBCT evaluation of grafting material and surrounding bone integration revealed promising results at 6 months post-operation, with the majority of both groups showing total union - 64.29% in the autogenous group and 57.14% in the xenogeneic group. These findings complemented the histomorphometry study of particulate dentin as a bone substitute, which observed 69.10% ± 22.80% direct contact between newly formed bone and grafted particles after 6 months [21].

This success rate paralleled outcomes achieved with allograft or xenograft in socket site preservation procedures. Furthermore, physiochemical studies have identified the release of BMP-2 - a protein crucial for osteoblast recruitment during bone formation - as a key factor. The gradual release of BMP-2 in amounts similar to allograft material from early stages may explain the comparable efficacy. The ATSG; tooth-derived bone substitute was reported continuously released BMP-2 and induced significant osteoblast migration [6]. Our previous study revealed that non-demineralized whole ATSG contains apatite with high crystallinity, known for its lower solubility compared to low crystalline apatite, suggesting its potential as an effective volume stabilizer in bone regeneration. This characteristic suggests ATSG's potential as an effective scaffold for bone regeneration [6].

Our present study supports these findings, as vertical and horizontal dimensional changes showed no significant differences between groups, which aligns with results from similar randomized clinical trials comparing autogenous demineralized dentin with inorganic bovine bone for alveolar bone defect augmentation. Furthermore, meta-analyses of marginal bone loss have demonstrated no statistically significant differences between autogenous dentin graft and xenogeneic bone graft at 6 months and final follow-up, indicating comparable effectiveness in preserving peri-implant bony structure for up to 18 months postoperatively [22]. It is important to acknowledge the limitations of this study, including no histologic data was collected. We evaluated and compared the quality and quantity of grafting materials clinically and radiographically. The local infection rate observed in our study (7.14%) aligns with findings from Barootchi et al.'s review on alveolar ridge preservation, which reported infection rates ranging from 2.80% to 9.10% during the first two postoperative weeks [23]. Despite ATSG's proven sterility following chemical treatment and decontamination protocols, infections occurred where grafting particles were present in the distal gingival crevice of second molars, combined with dental plaque accumulation. These cases resolved with particle removal, irrigation, and antibiotics. No significant graft loss was noted, though healing took slightly longer than usual. Graft exposure due to wound dehiscence was also observed but did not affect bone levels, as only a few particles were clinically visible and removed. The influence of age on postoperative outcomes has been well-documented, with multiple studies indicating higher postoperative morbidity - including pain, swelling, and local infection - following third molar removal in patients over 25 years. Research has shown that postoperative pocket depth increases occurred in 51.00% of patients older than 26 years, with a large-scale study of 4,004 patients demonstrating 1.5 times higher complication rates in patients over 25 years of age. [24]. Our study's narrow age range (20-28 years, mean 23.10 ± 2.70) might be one factor causing consistent healing patterns observed across participants. This age-related consideration emphasized the importance of accounting for patient age in surgical planning for bone grafting procedures.

 In conclusion, ATSG demonstrated clinical efficacy comparable to xenogeneic material in alveolar ridge preservation. The here presented approach offers multiple advantages over commercially available bone substitute materials: it provides an efficient method for upcycling medical waste, represents a viable autogenous grafting option without donor site morbidity, and can be prepared chairside within minutes. These findings support ATSG as a valuable addition to the range of grafting materials available for clinical use in alveolar ridge preservation, while the performance of ATSG in other regeneration procedures of the alveolar ridge remains yet to be elucidated.

## Electronic supplementary material

Below is the link to the electronic supplementary material.

Supplementary Material 1

## Data Availability

No datasets were generated or analysed during the current study.
