# Supplementary material for: Comparable efficacy of autogenous tooth-derived grafts and xenografts in post-extraction osseous defects: a split-mouth randomized trial
Source: Clin Oral Investig. 2025 Jul 23;29(8):388. doi: 10.1007/s00784-025-06458-3 (PMC12287208; doi:10.1007/s00784-025-06458-3)
Supplement: Supplementary file 1 — Supplementary Material 1 [file 784_2025_6458_MOESM1_ESM.docx]

**Supplemental data**

**Mean grey value and fractal analysis**

Standard periapical films were taken by a single experienced technician. The intraoral film's anterior border was positioned at the first mandibular molar's mesial crown aspect, ensuring visualization of the second molar's distal root apex and alveolar bone. Radiographs were digitized and saved in JPEG format. ROIs were selected following the protocol below. ImageJ 2.1.0 software was used to calculate mean grey values (indicating bone density) and perform fractal analysis of each ROI.


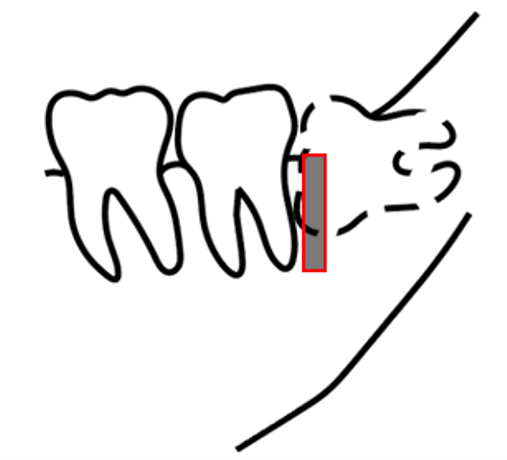


**Supplemental figure 1**: Region of interest (ROI) in MGV evaluation and fractal analysis

The area was created by drawing a box approximating the periodontal space of the distal root of the mandibular second molar. In the supero-inferior dimension, the vertical border of the box extended from the cemento-enamel junction (CEJ) to the apex of the distal root of the mandibular second molar, with a width of 2 mm.

**Grafted material and bone integration evaluation**

We evaluated the central groove of the second mandibular molar in sagittal, axial, and coronal planes. The integration between grafted material and bone was then scored according to the criteria below.

**
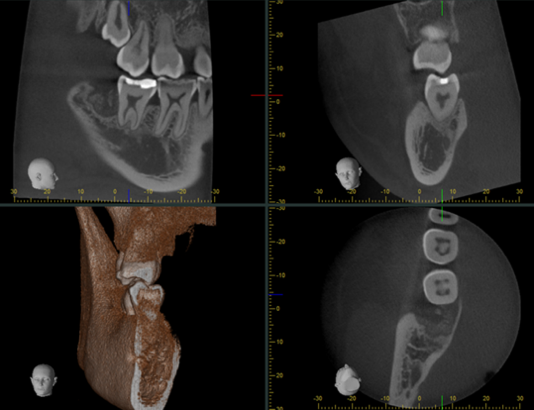
**

**Score 0** (Non-union): Radiolucent line present along the entire margin between grafted material and bone.


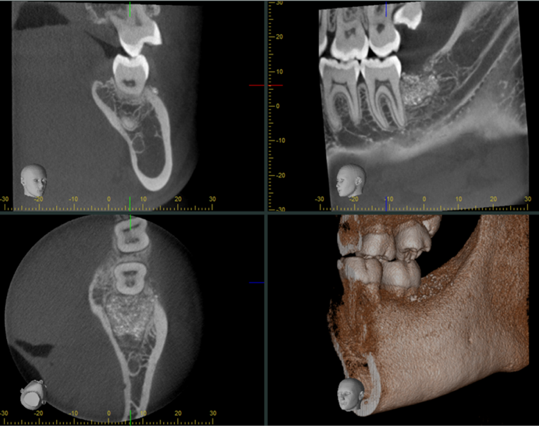


**Score 1** (Partial union): Radiolucent line partially present along the margin between grafted material and bone.


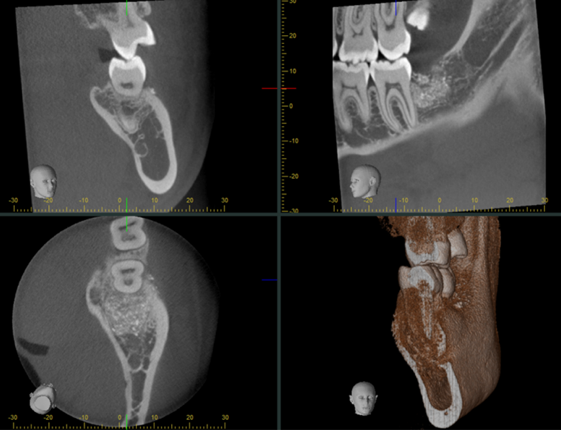


**Score 2** (Total union): No radiolucent line present along the margin between grafted material and bone.

**Vertical bone dimension measuring**

Vertical bone dimension was a parameter which defined whether there was a bone gain or bone loss vertically after the graft placement. The vertical bone dimension was evaluated using cone beam computed tomography (CBCT). The measurement was performed at the central groove of the second mandibular molar in the sagittal plane. The occlusal plane of the second mandibular molar was rotated parallel to the horizontal plane before taking measurements.

**
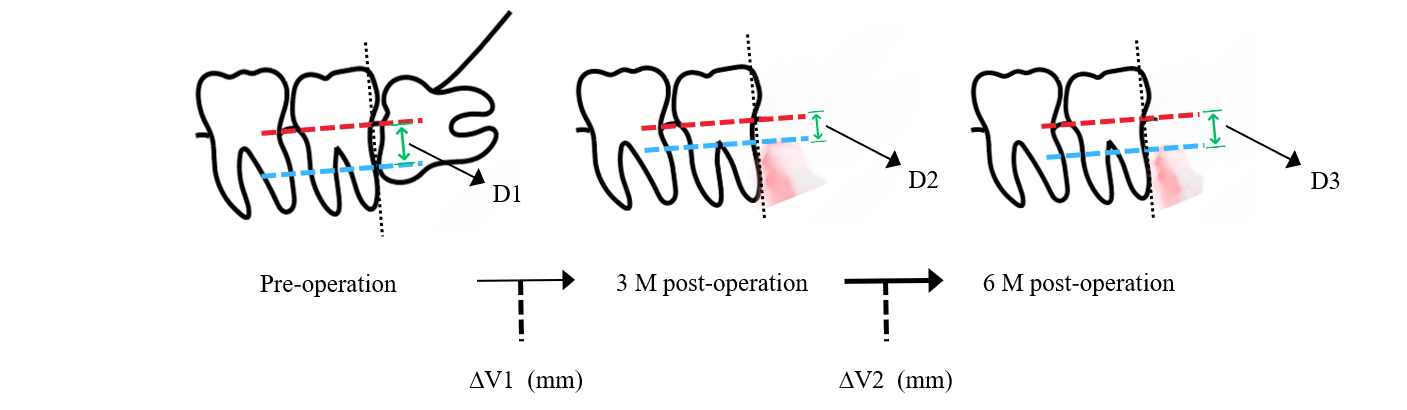
**

**Supplemental figure 2**: measurement of vertical bone dimension at the grafting areas

D1, D2 and D3 are distances measured from the highest point of alveolar bone or grafting material to the CEJ of distal aspect of 2^nd^ mandibular molars

Δ V1 is a vertical dimension change from pre-operation and 3 months post-operation.

Δ V2 is a vertical dimension change from 3 and 6 months post-operation

**Bucco-lingual bone width measuring**

Horizontal bone dimension was a parameter which defined whether there was a bone formation or bone resorption bucco-lingually after the graft placement. The bucco-lingual bone width was evaluated in the axial plane using cone beam computed tomography (CBCT)**.** The occlusal plane of the second mandibular molar was rotated parallel to the horizontal plane. The measurement level was determined at the highest point of the grafting material, as observed in sagittal plane at 3 months post-operation.


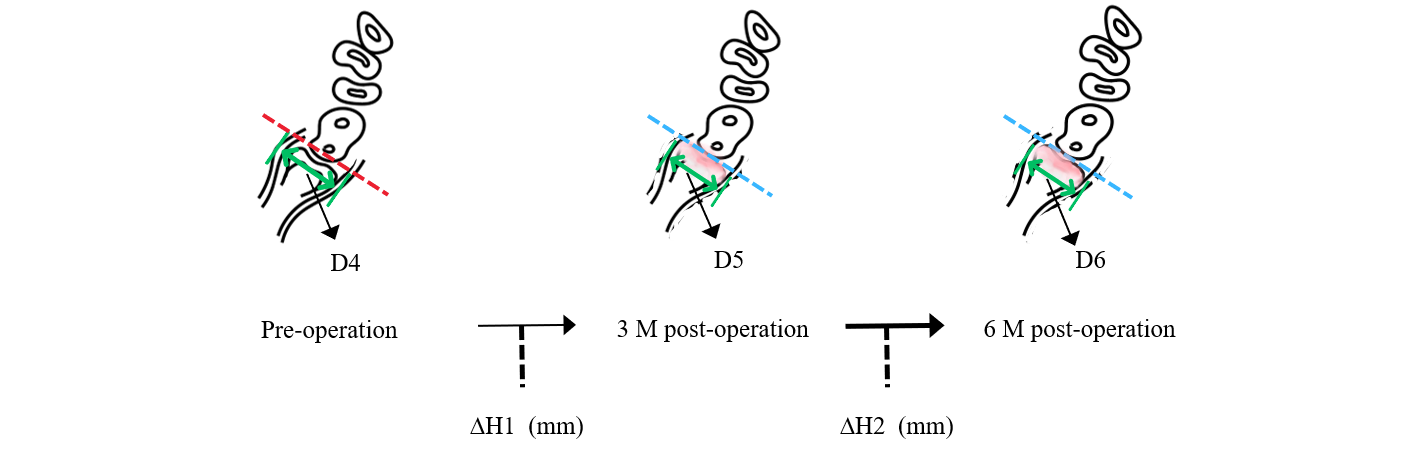
**Supplemental figure 3: measurement of horizontal bone dimension at the grafting areas**

D4, D5 and D6 are distances measured from the bucco-lingual width at distal aspect of 2^nd^ mandibular molar

Δ H1 is a horizontal dimension change from pre-operation and 3 months post-operation.

Δ H2 is a horizontal dimension change from 3 and 6 months post-operation

| **Supplemental table 1: Mean +/- SD of probing depth of the second molar at different time points** | | | | | | | | | |
| --- | --- | --- | --- | --- | --- | --- | --- | --- | --- |
|  | **Disto-Buccal Probing Depth Means** | | | **Mid-Distal Probing Depth Means** | | | **Disto-Lingual Probing Depth Means** | | |
|  | Graft Autogenous | Graft Xenogeneic | p-value | Graft Autogenous | Graft Xenogeneic | p-value | Graft Autogenous | Graft Xenogeneic | p-value |
| Pre-op | 4.357 +/- 2.20^a^ | 3.786 +/- 1.12^a^ | .319 | 3.429 +/- 0.65^a^ | 3.786 +/- 1.58 ^a, b^ | .418 | 3.929 +/- 1.33 ^a^ | 3.643 +/- 1.22 ^a^ | .414 |
| 2 weeks | 6.071 +/- 2.68^a^ | 6.357 +/- 1.86^b^ | .659 | 5.071 +/- 2.43^a^ | 5.929 +/- 2.59 ^a^ | .152 | 4.357 +/- 2.34 ^a^ | 4.071 +/- 1.49 ^a^ | .682 |
| 3 months | 4.286 +/- 1.68^a^ | 3.571 +/- 0.94^a^ | .096 | 4.000 +/- 1.84^a^ | 3.857 +/- 1.29^b^ | .671 | 3.143 +/- 0.54 ^a^ | 3.000 +/- 0.68 ^a^ | .336 |
| 6 months | 3.429 +/- 1.65^a^ | 4.000 +/- 1.84^a^ | .104 | 4.000 +/- 1.84^a^ | 3.214 +/- 1.37 ^b^ | .222 | 3.000 +/- 0.55 ^a^ | 2.929 +/- 0.83 ^a^ | .775 |

**Tooth mobility of the second mandibular molars at before and after material grafting.** (Noted that only first-degree mobility was observed throughout the study.)

|  | **Graft Autogenous** | **Graft Xenogeneic** | **p-value** |
| --- | --- | --- | --- |
| Pre-op, n (%) | 0 (0.0%) | 1 (7.1%) | - |
| 2 weeks, n (%) | 3 (21.4%) | 3(21.4%) | .750^a^ |
| 3 months, n (%) | 5(35.7%) | 4(28.6%) | .500^a^ |
| 6 months, n (%) | 0(0.0%) | 0(0.0%) | - |

| a, p-value from McNemar test  n, surgical site |  |  |  |  |
| --- | --- | --- | --- | --- |
